# The small screw-apex distance is potentially associated with femoral head osteonecrosis in adults with femoral neck fractures treated by closed reduction and percutaneous 3 parallel cannulated screws

**DOI:** 10.1186/s12891-024-07380-7

**Published:** 2024-04-13

**Authors:** Xiaoxiao Zhou, Shengyang Guo, Wenjun Pan, Linyuan Zhang, Houlin Ji, Yang Yang

**Affiliations:** 1 Department of Orthopedics, Jiangwan Hospital of Hongkou District of Shanghai, Shanghai, China; 2 Department of Orthopedics, Shanghai University of Medicine & Health Sciences Affiliated Zhoupu Hospital, Shanghai, China; 3 Department of Orthopedics, Taizhou Hospital of Zhejiang Province, Affiliated to Wenzhou Medical University, No. 150 Ximen Street, Linhai City, 317000 Zhejiang Province China; 4 Jinji Lake Community Health Service Center of Suzhou Industrial Park, Jiangsu, 215000 China

**Keywords:** Femoral neck fracture, Osteonecrosis, Cannulated screws, Screw-apex distance, Weight-bearing area

## Abstract

**Objective:**

Femoral neck fractures (FNFs) are among the most common fractures in elderly individuals. Surgery is the main treatment for FNFs, and osteonecrosis of the femoral head (ONFH) is one of the unacceptable complications. This study aimed to assess both the clinical and radiological outcomes in patients with FNFs treated with three parallel cannulated screws and to identify relationship between screws position and ONFH.

**Patients and methods:**

A total of 100 patients who were treated with closed reduction and fixed with 3 parallel cannulated screws met the inclusion criteria between January 2014 and December 2020 at authors’ institution. The follow-up duration, age, sex, affected side, and injury-to-surgery interval were collected; the neck-shaft angle of both hips, screw-apex distance (SAD) and the tip-apex distance (TAD)were measured; and the Garden classification, quality of reduction and presence of ONFH were evaluated.

**Results:**

The sample consisted of 37 males and 63 females, with 60 left and 40 right hips affected. The mean age of patients was 54.93 ± 12.24 years, and the mean follow-up was 56.3 ± 13.38 months. The overall incidence of ONFH was 13%. No significant difference was observed in the incidence of ONFH by affected side, age, fracture displacement, injury-to-surgery interval, neck-shaft angle deviation, or reduction quality. The SAD was significantly shorter in ONFH patients than in normal patients for all three screws (*p* = 0.02, 0.02, and 0.01, respectively).

**Conclusions:**

The short SAD of all screws is associated with femoral head necrosis of FNFs treated with 3 cannulated screws. The short SAD indicated that screws malpositioning in the weight-bearing area of the femoral head, potentially harming the blood supply and compromising the anchorage of the primary compressive trabeculae in this region.

## Introduction

Femoral neck fractures (FNFs) are among the most common fractures in elderly individuals, accounting for approximately 50% of all hip fractures [1]. These fractures pose a serious threat to the health and quality of life of patients [2–4]. Surgery is the main treatment for FNFs, as it allows patient mobilization and reduces the risk of complications. A variety of implants, including cannulated screws, dynamic hip screws, full-thread headless compression screws, the Targon system, and hip prostheses, femoral neck system are used in FNF treatment [5–7]. Cannulated screw fixation is the classical treatment option for FNFs because of its advantages, such as minimal invasiveness, reduced operative time, and sufficient stability [8]. However, the optimal positioning and volume of screws in the femoral neck remain controversial [9]. Traditionally, three cannulated screws are implanted in a parallel inverted triangle configuration [10]. This configuration provides the advantage of allowing compression within the fracture gap and enabling some collapses during the fracture heals, which has been demonstrated to be biomechanically superior to other implants [11].

Complications of this treatment, including postoperative nonunion, screw cut-out or fixation failure, femoral neck shortening, and osteonecrosis of the femoral head (ONFH), have been reported. ONFH is a severe and prevalent postoperative complication of FNFs. ONFH is thought result from the impairment of the unique blood supply to the femoral head. The overall incidence of ONFH ranges from 14.8–30% [12, 13], and it frequently manifests 2–3 years after treatment [14]. Although numerous studies have focused on factors influencing ONFH, its underlying pathophysiological mechanism remains incompletely understood. Independent risk factors, such as chronic disease, delayed surgery, fracture displacement, age, and sex, remain controversial [15, 16]. Furthermore, the potential impact of screw spatial positioning on the development of ONFH has not been investigated. The aim of this retrospective study was to assess both the clinical and radiological outcomes of FNF patients who underwent closed reduction and fixation with three parallel cannulated screws and to identify relationship between screws position and ONFH.

## Patients and methods

### Study Population

We retrospectively reviewed the records of patients who underwent surgical treatment for FNFs between January 2014 and December 2020 at our institutions. This study was approved by the ethics committees of the participating hospitals. The inclusion criteria included: (1) patients diagnosed FNFs treated with closed reduction and surgical placement of 3 parallel cannulated screws; (2) patients aged over than 18 years at the time of injury; (3) availability of complete clinical and radiological data; and (4) a minimum follow-up duration of 2 years. The exclusion criteria included: (1) fracture treated with an internal fixation construct other than three parallel cannulated screws, including fully threaded parallel conical screws, four cannulated screws with a cross screw, and a femoral neck system (FNS); (2) fracture treated with open reduction or over 2 weeks after injury; (3) pathological fracture; (4) concomitant systemic immune disease requiring long-term steroid use or alcoholism; (5) follow-up duration shorter than two years; and (6) insufficient clinical data or incomplete radiographs.

Finally, a total of 100 patients treated by closed reduction and 3 parallel cannulated screws met the inclusion criteria. The cohort were divided into ONFH and normal groups.

### Radiographic measurements

**Fig. 1:**
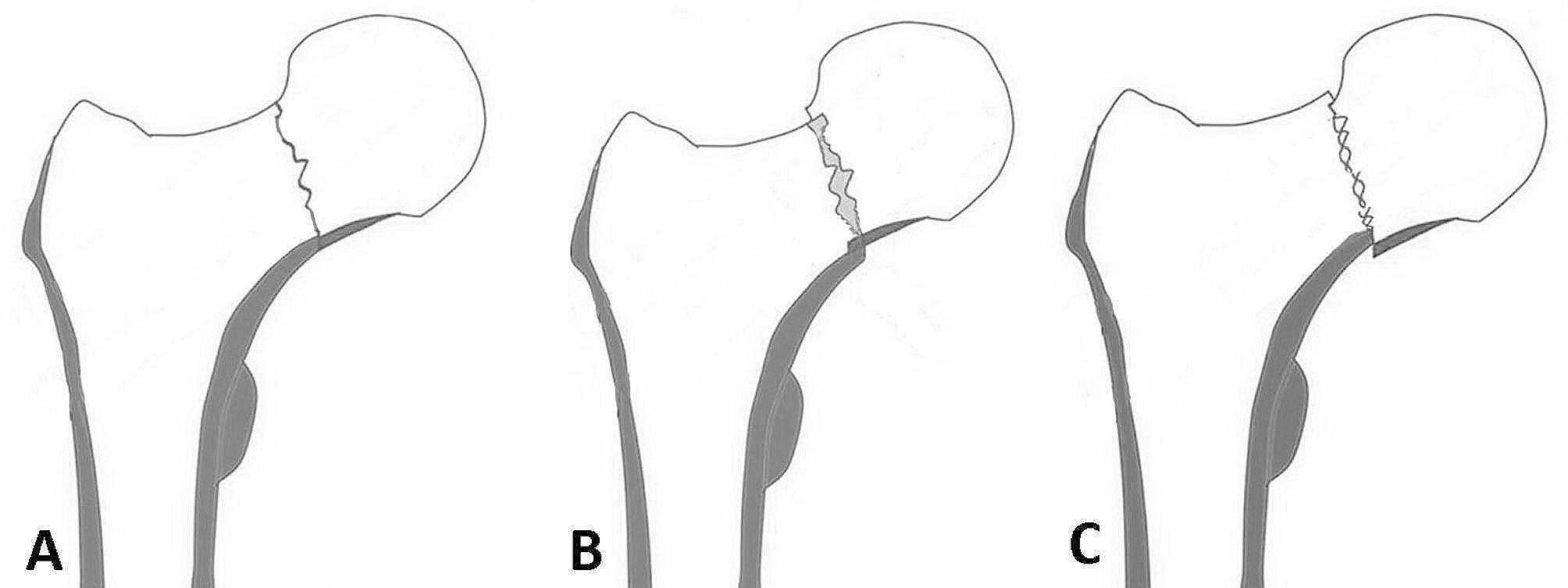
Schematic diagram showing the quality of femoral neck fracture reduction, including neutral support (anatomic reduction) of the fracture fragment (**A**), positive support (**B**), and negative support (**C**). Positive support indicates medial cortical support of the distal edge of the femoral neck fragment (**B**), and negative support indicates no medial cortical support of the distal edge (**C**)

Anteroposterior (AP) and lateral radiographs of the hip were obtained to measure parameters at the first month of followup. The neck-shaft angle of both hips, the screw-apex distance (SAD) and the tip-apex distance (TAD) were measured using a Picture Archiving and Communication System (PACS; GE Healthcare, Chicago, Illinois). The Garden classification, and the quality of reduction were assessed, which included the presence of neutral support (anatomical reduction), positive support, and negative support (Fig. 1A-C) [17], and the presence of ONFH was evaluated by two experienced orthopaedic surgeons (XXZ and SYG). At each follow-up appointment, AP and lateral radiographs were routinely obtained for monitoring complications. ONFH was characterized by the emergence of subchondral osteolysis, accompanied by cystic alterations or sclerosis, and the occurrence of segmental collapse as evident on postoperative radiographs. However, patients continued to experience persistent hip pain and an inability to bear weight [18–22].

**Fig. 2:**
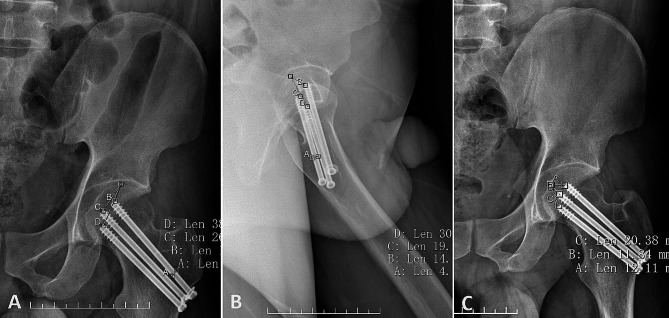
Schematic diagram showing the definition of the screw-apex distance (SAD) and the tip-apex distance (TAD). The SAD was measured as the distance between the apex of the weight-bearing area and the end of each screw on anteroposterior (AP) and conventional lateral radiographs of the hip (**A** and **B**). The TAD was measured as the distance between the apex of the medial femoral head and the tip of each screw on AP and lateral radiographs of the hip (**C** and **B**). All images were calibrated using the known diameter of the screw

The SAD of the weight-bearing area was defined as the distance between the apex and the end of each screw, measured on both AP and lateral radiographs of the hip (Fig. 2A and B). The TAD of the medial femoral head was defined as the distance between the fossa capitis femoris and the tip of each screw, measured on both AP and lateral radiographs of the hip(Fig. 2B and C) [23]. The known diameter of the cannulated screws was used for calibrating all parameter measurements. On AP radiographs of the hip, the screw closest to the apex of the weight-bearing area was defined as the 1st screw, followed sequentially by the 2nd and 3rd screws.

### Closed reduction and percutaneous screw fixation

Surgery was performed promptly after diagnosis, typically within 24 h, by experienced orthopaedic surgeons. The patient was positioned in a supine position on a speciallized orthopaedic traction bed. The affected lower limb was initially abducted and externally rotated to enlarge the space of fractures, then adducted and internally rotated under longitudinal traction for reduction until the patella oriented horizontally. The reduction quality was verified in the AP and lateral planes with a cross-table view using a C-arm X-ray machine. After reduction, three Kirschner wires were usually used as guide pins, and cannulated screws were percutaneously inserted using a freehand technique. The primary goal was Neutral support (anatomical reduction), with positive support as the secondary aim. Negative support was also deemed acceptable if stable reduction of the fracture has been achieved (Fig. 1A-C). Sometimes, a Kirschner wire was inserted into the femoral head, serving as a joystick to control the head to assist the reduction. Subsequently, three partially threaded parallel cannulated screws (φ = 6.5 mm, Smith & Nephew, UK) were placed; the first implant was inserted along the femoral calcar (inferior), and the other two were placed superiorly (posterosuperior, anterosuperior) to form an inverse triangular configuration. The choice between a regular or an inverse triangle configuration was at the surgeon’s discretion.

### Rehabilitation and follow-up

All patients followed a standardized postoperative protocol, which included the following: refraining from putting weight on the affected limb for the initial 3 months, transitioning to partial weight-bearing after bone union, and advancing to full weight-bearing after 6 months post-operation [24]. The following demographic and clinical data were collected: follow-up duration; age; sex; affected side; and injury-to-surgery interval.

### Statistical analysis

Statistical analysis was conducted using SPSS 23.0 software (SPSS, Inc., IBM Corporation, Armonk, NY, USA). Continuous variables were presented as the mean ± standard deviation. The independent-samples *t*-test was used to compare differences between the ONFH group and the normal group. Categorical variables, presented as numbers and percentages, were anlyzed using the chi-square test or Fisher’s exact test when the expected count was less than 5. The significance level was set at 0.05.

## Results

This sample consisted of 37 males and 63 females. The mean patient age was 54.93 ± 12.24 (23 to 82) years, and the mean follow-up duration was 56.3 ± 13.38 (27 to 72) months. This study involved 60 left hips and 40 right hips; 34 patients presented with nondisplaced Garden class I–II FNFs, and 66 patients with displaced Garden class III–IV FNFs. The overall incidence of ONFH was 13% (13/100). The mean time of fracture to ONFH was 27.75 ± 10.24 months (range, 13 to 46 months), with all cases of ONFH occurring between 1 and 4 years after surgery.

There was no significant difference between the ONFH and normal groups with regard to affected side, age, displacement (Garden classification), injury-to-surgery interval, neck-shaft angle deviation, or reduction quality. In the ONFH group, there were 13 females and no males, with a significant difference in the incidence of ONFH observed between male and female patients (*p* < 0.01) (Table 1).

**Table 1:** Summary of the main demographic data of the two groups

Basic characteristics	ONFH patients (*n* = 13)	Normal patients (*n* = 87)	P value
Age(years)	58.38 ± 11.03 (38–78)	54.74 ± 14.35 (23–82)	0.38
Sex			
Male(n, %)	0(0%)	37(42.5%)	< 0.01
Female(n, %)	13(100%)	50(57.5%)	
Operative side			0.56
Left(n, %)	9(69.2%)	51(58.6%)	
Right(n, %)	4(30.8%)	36(41.4%)	
Garden classification			0.53
I + II(n, %)	3(23.1%)	31(35.6%)	
III + IV(n, %)	10(76.9%)	56(64.4%)	
Reduction quality			0.32
negative(n, %)	3(23.1%)	8(9.2%)	
positive(n, %)	2(15.4%)	13(14.9%)	
neutral support(n,%)	8(61.5%)	66(75.9%)	
Injury-to-surgery interval (days)	2.38 ± 1.04 (1–4)	2.80 ± 1.85 (1–14)	0.43
Follow-up duration (months)	59.38 ± 13.47 (29–72)	55.84 ± 13.38 (27–72)	0.38
Deviation of neck-shaft angle (°)	1.74 ± 6.97 (-12.13-13.63)	4.33 ± 7.68 (-11.41-20.94)	0.26

ONFH, osteonecrosis of the femoral head. Continuios data are presented as the mean ± standard deviation(range) and categorical data are present as percentage. The significance level was set at 0.05

The TAD of the 1st screw and the 1st screw plus 2nd screw in AP view were significantly longer in ONFH group, compared to the normal group(*p* < 0.001, *p* < 0.01, respectively). No significant difference was found regarding of the sum value of the 1st, 2nd and 3rd screws in the AP view. The cumulative TAD values in both AP view and lateral view showed no significant difference between the ONFH group and the normal group (Table 2).

**Table 2:** Comparison of the TAD between the two groups

	AP view			Lateral view			TAD		
Items	ONFH group (*n* = 13)	Normal group (*n* = 87)	*P* value	ONFH group (*n* = 13)	Normal group (*n* = 87)	*P* value	ONFH group (*n* = 13)	Normal group (*n* = 87)	*P* value
1st screw(mm)	21.35 ± 5.46 (14.28–29.13)	15.95 ± 4.52 (5.02–28.42)	< 0.001	13.13 ± 5.80 (6.60-21.79)	14.90 ± 4.73 (1.88–28.16)	0.22	34.48 ± 6.36 (23.07–43.04)	30.85 ± 7.36 (13.51–53.29)	0.10
1st + 2nd screw(mm)	37.51 ± 6.59 (25.66–51.38)	31.70 ± 6.42 (22.34–55.48)	< 0.01	31.06 ± 11.05 (19.40-54.54)	34.17 ± 9.18 (3.96–61.51)	0.27	68.57 ± 14.00 (47.93–94.40)	65.87 ± 13.01 (33.71-106.14)	0.49
1st + 2nd + 3rd screw(mm)	56.99 ± 9.00 (43.34–70.90)	52.13 ± 9.86 (32.69–87.20)	0.10	52.84 ± 16.76 (34.63–89.35)	58.36 ± 14.19 (6.37–96.91)	0.20	109.81 ± 20.29 (80.00-160.25)	110.49 ± 20.73 (60.26-164.33)	0.91

ONFH, osteonecrosis of the femoral head; TAD, tip-apex distance; AP, anteroposterior

Data are presented as the mean ± standard deviation(range) or number of patients. Differences are considered significant at *p* < 0.05

The SAD, the distance from the tip of the screw to the apex in the weight-bearing area of the femoral head, was significantly shorter in the ONFH group compared to the normal group. Specifically, the SAD of the 1st screw in the ONFH group(13.48 ± 6.73 mm; range, 4.54–31.77 mm) was significantly shorter than in the normal group (18.47 ± 6.49 mm; range, 3.12–36.63 mm) in AP radiographs. Similar trends were observed for the combined SAD of the 1st and 2nd screws (*p* = 0.01), as well as the 1st, 2nd, and 3rd screws combined (*p* = 0.01), as indicated in AP radiographs (Table 3).

**Table 3:** Comparison of the SAD between the two groups

	AP view			Lateral view			SAD		
Items	ONFH group (*n* = 13)	Normal group (*n* = 87)	*P* value	ONFH group (*n* = 13)	Normal group (*n* = 87)	*P* value	ONFH group (*n* = 13)	Normal group (*n* = 87)	*P* value
1st screw(mm)	13.48 ± 6.73 (4.54–31.77)	18.47 ± 6.49 (3.12–36.63)	0.01	13.13 ± 5.80 (6.60-21.79)	14.90 ± 4.73 (1.88–28.16)	0.22	26.61 ± 10.32 (11.73–53.56)	33.37 ± 9.33 (12.13–60.70)	0.02
1st + 2nd screw(mm)	34.95 ± 13.52 (13.78–70.49)	44.80 ± 12.44 (17.10-77.86)	0.01	31.06 ± 11.05 (19.40-54.54)	34.17 ± 9.18 (3.96–61.51)	0.27	66.01 ± 21.86 (34.79-125.02)	78.97 ± 17.68 (43.96-130.26)	0.02
1st + 2nd + 3rd screw(mm)	67.38 ± 18.80 (40.10-113.63)	81.54 ± 17.66 (40.81-128.83)	0.01	52.84 ± 16.76 (34.63–89.35)	58.36 ± 14.19 (6.37–96.91)	0.20	120.20 ± 30.03 (77.60-202.98)	139.90 ± 25.87 (87.73-217.02)	0.01

ONFH, osteonecrosis of the femoral head; SAD, screw-apex distance; AP, anteroposterior

Data are presented as the mean ± standard deviation(range) or number of patients. Differences are considered significant at *p* < 0.05

Although there were no significant differences in these distances in lateral radiographs between two groups (*p* = 0.22, 0.27, and 0.20, respectively), the corresponding total SADs of the three screws in AP and lateral radiographs were significantly shorter in the ONFH group than in the normal group (*p* = 0.02, 0.02, and 0.01, respectively) (Table 3).

## Discussion

The most significant finding of this study was the markedly shorter SAD of the first screw, the combined first screw and second screw, and the sum of all three screws in the ONFH group compared to the normal group. Conversely, the TAD of the first screw, the combined first screw and second screw, and the sum of all three screws in the ONFH group showed no significant difference when compared to the normal group. A reduced SAD implies that the screw tip is closer to the weight-bearing area of the femoral head, potentially correlating with ONFH in femoral neck fractures treated with three cannulated screws.

Multiple factors have been identified as contributors to ONFH, including fracture displacement, segmental rotation, delayed time to surgery, postoperative malposition, and fracture patterns [15, 25, 26]. Achieving satisfactory reduction and rigid fixation is crucial in reducing the incidence of ONFH. Although various fixation devices are currently available, the use of three cannulated screws in a inverted triangle configuration is the most prevalent method in FNF treatment, owing to its excellent mechanical strength, rigid fixation, minimal invasiveness, and technical simplicity [27, 28]. The precise insertion of the three screws were key point to avoid ONFH.

The pathophysiological mechanisms underlying osteonecrosis of ONFH have been a subject of debate for many years, with significant gaps in understanding its pathogenesis yet to be addressed. There is a strong argument for vascular hypotheses in the aetiology of ONFH, which posit that a reduction in blood perfusion within the femoral head is critical to its pathogenesis [29]. The lateral epiphyseal vessels are of paramount importance, accounting for vascular supply to 80% of the proximal and weight-bearing areas of the femoral head [30, 31]. Their vulnerability to damage, compression, or occlusion positions these vessels as central in the pathogenesis of osteonecrosis, particularly in areas anatomically prone to such conditions, like the anterosuperior segment of the femoral head [32]. It has been argued that the force of the initial trauma determines the fate of vascularity in these fractures and the development of ONFH [33]. Furthermore, stress on the retinacular vessels has been identified as a primary factor in the development of ONFH [34], with the absence of these vessels being a significant contributor to its pathogenesis [35]. In displaced FNFs, tearing or kinking of these vessels can severely limit blood supply, potentially leading to nonunion and ONFH [36]. Consequently, the epiphysis and articular surface are especially prone to circulatory insufficiency [37], especially in weight-bearing areas. A digital subtraction angiography study revealed that vessel damage rates in Garden class III and IV fractures could reach 72.7-100% [38]. Nonetheless, 95% of patients in Garden class III and 80% in Garden class IV retained intact superior retinacular artery blood supplies, without showing signs of ischemia [39]. Another study suggested that higher Garden classifications do not necessarily correlate with increased ONFH risk [25], and some researchers have postulated that intraosseous healing may compensate for part of the devascularised head [40].

**Fig. 3:**
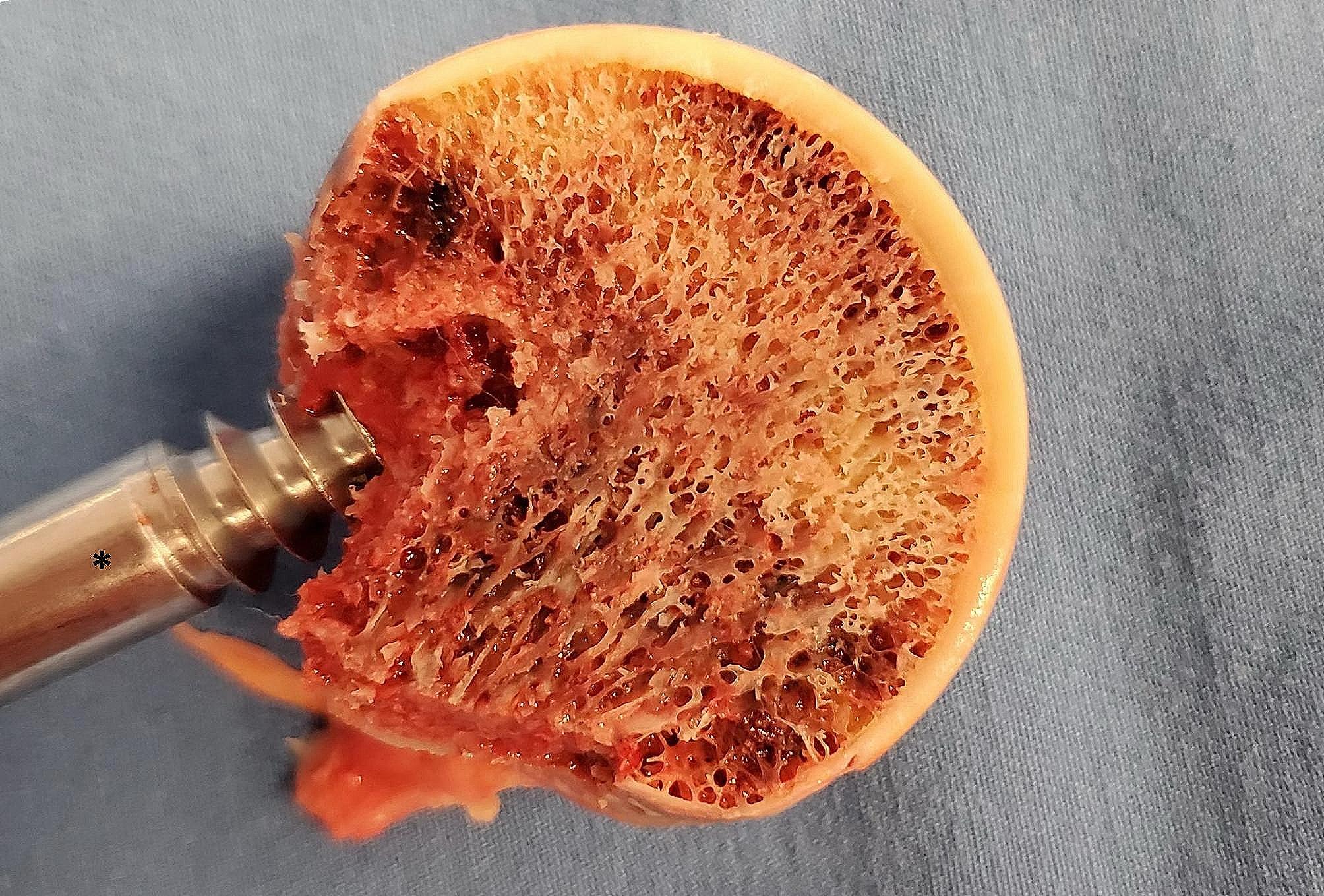
An 81-year-old female patient suffering from a right femoral neck fracture was treated with artificial hip replacement. The femoral head was cut with a saw near the centre on the coronal plane after the femoral head was removed, with the weight-bearing region just above the femoral head. Here, we aim to show the principal compressive trabecular structure. * Extractor

The proximal femur comprises five trabecular groups, displaying highly asymmetric distribution, which may lead to diverse pathomechanisms [41]. In the femoral head’s upper and central areas, principal compressive trabeculae are prominent, transmitting weight from the articular surface to the neck and fulcrum (Fig. 3). The principal tensile trabeculae, which exhibit a more horizontal orientation, intersect and extend into the lower medial, non-weight-bearing region [42]. Research suggests that both the elastic modulus and functions of these trabeculae are comparable to cortical bone [43]. Trabecular degeneration has been identified as a crucial factor in the incidence of hip fragility fractures, predominantly occurring in the femoral neck and intertrochanteric regions [44]. However, the exact role of trabecular injury in ONFH remains unclear.

As delineated by the vascular pathophysiological mechanisms, the femoral head endures both static and dynamic weight-bearing biomechanical demands, resulting in uneven distribution of mechanical forces on its surface [45]. Notably, areas subjected to maximal loading demonstrate a discernible 25% increase in vessel density within the subchondral bone [46]. Research has further investigated the relationship between mechanical loading and bone perfusion, indicating that regions subjected to increased mechanical stress exhibit reduced perfusion [47]. This indicates that trabeculae located under the weight-bearing region need more blood supply and demonstrate increased sensitivity to ischemia. According to our results, a higher incidence of ONFH was associated with a shorter SAD, the tips of the screws were closer to the apex of the superolateral aspect of the femoral head. Consequently, FNFs-induced damage to the retinacular vessels would exacerbate the circulatory insufficiency, especially in the epiphysis and weight-bearing area. Meanwhile, damage to the screw anchorage in the principal compressive trabeculae on the articular surface may be another important factor. Such damage could reduce the structural integrity of the anchorage, leading to subchondral microfractures, accompanied by a minor extent of bone cell death, thereby impairing normal reparative processes in the femoral head. This impairment, along with osteocytes ischemic necrosis, could eventually lead to structural collapse of the femoral head. However, the collapse phenomenon can be further explicated by the imbalance between the slower rate of bone formation and the more rapid resorption rate in the subchondral bone, resulting in a net bone loss, compromised structural integrity, and eventual subchondral fracture [48].

This study’s limitations include its small sample size and retrospective design. Additionally, the monocentric data collection and absence of randomization introduce potential for selection bias. The short follow-up duration in this study, although the mean follow-up was 56.3 ± 13.38 months, may underestimate the actual incidence of ONFH. Asnis et al. reported ONFH in 11% of their patients after two years, increasing to 22% at eight years, and almost 25% of their patients had nondisplaced Garden I or II fractures [41]. Additionally, the use of radiographs and CT scans to diagnose osteonecrosis likely leads to further underreporting. Magnetic resonance imaging (MRI) is more sensitive for detecting osteonecrosis, but postoperative MRI is not routinely performed. Additionally, hip rotation during measurements can introduce significant errors in screw positioning [9], especially in TAD measurements. The absence of data on nonunion, screw cut-out or fixation failure, and femoral neck shortening also represents a notable limitation. However, despite these limitations, we believe that our findings are robust and provide empirical evidence that the spatial malpositioning of screws may increase the risk of ONFH in FNFs patients.

## Conclusions

In conclusion, the short SAD of all screws is potentially associated with femoral head necrosis of FNFs treated with 3 cannulated screws. The short SAD indicated that screws malpositioning in the weight-bearing area of the femoral head, which may result in harm to the unique blood supply and compromise the anchorage of the primary compressive trabeculae in this region. This damage may lead to ischaemic necrosis of bone cells and loss of structural integrity of the principal compressive trabeculae, eventually leading to structural collapse of the femoral head.

## Data Availability

All data will be available upon motivated request to the corresponding author of the present paper.

## Abbreviations

FNFs: Femoral neck fractures
ONFH: osteonecrosis of the femoral head
SAD: screw-apex distance
TAD: tip-apex distance
AP: anteroposterior
MRI: Magnetic resonance imaging
